# The global fight against *trans*-fat: the potential role of international trade and law

**DOI:** 10.1186/s12992-019-0488-4

**Published:** 2019-07-11

**Authors:** Andrea Parziale, Gorik Ooms

**Affiliations:** 1 Sant’Anna School of Advanced Studies, Pisa, Italy; 2 Global Health & Development, London School of Hygiene & Tropical Medicine (UK), Keppel St, Bloomsbury, London, WC1E 7HT UK

**Keywords:** Global health governance, Public health, World Health Organization, Non-communicable diseases, *trans*-fat, International trade, International law

## Abstract

Non-communicable diseases in general and cardiovascular diseases in particular are a leading cause of death globally. *Trans*-fat consumption is a significant risk factor for cardiovascular diseases. The World Health Organization’s ‘REPLACE’ action package of 2018 aims to eliminate it completely in the global food supply by 2023. Legislative and other regulatory actions (i.e., banning *trans*-fat) are considered as effective means to achieve such a goal. Both wealthier and poorer countries are taking or considering action, as shown by the United States food regulations and Cambodian draft food legislation discussed in this paper. This paper reviews these actions and examines public and private stakeholders’ incentives to increase health-protecting or health-promoting standards and regulations at home and abroad, setting the ground for further research on the topic. It focuses on the potential of trade incentives as a potential driver of a ‘race to the top’. While it has been documented that powerful countries use international trade instruments to weaken other countries’ national regulations, at times these powerful countries may also be interested in more stringent regulations abroad to protect their exports from competition from third countries with less stringent regulations. This article explores practical and principled considerations on how such a dynamic may spread *trans*-fat restrictions globally. It argues that trade dynamics and public health considerations within powerful countries may help to promote anti-*trans*-fat regulation globally but will not be sufficient and is ethically questionable. True international regulatory cooperation is needed and could be facilitated by the World Health Organization. Nevertheless, the paper highlights that international trade and investment law offers opportunities for anti-*trans*-fat policy diffusion globally.

## Introduction

International and national public health authorities are aware that the global fight against non-communicable diseases (NCDs) requires addressing their social and commercial determinants. The consumption of unhealthy food is the result of wider economic and legal arrangements and corporate strategies, not only a matter of individual life-style choices. In May 2018, the World Health Organization (WHO) released the ‘REPLACE’ action package [1], calling on governments to remove *trans*-fats from the global food chain by 2023. REPLACE claims that eliminating such a harmful substance from processed foods could prevent hundreds of thousands of heart attacks and deaths annually. It also argues that the most effective tool to reduce artificial *trans*-fat in the food supply and individual consumption seems to be legislative or other regulatory action aimed at limiting or prohibiting industrially produced *trans*-fat.

REPLACE signals that the fight against industrially produced *trans*-fat has reached the global agenda and that regulatory action is considered to be key to removing such a harmful substance from the food supply chain. In this paper, we will explore whether and how trade dynamics may encourage the global spread of *trans*-fat restrictions. To our knowledge, while the existent trade and health literature focuses on how transnational corporations, backed by powerful states, lobby to weaken food regulation internationally [2–4], the opposite – how the interests of public and private stakeholders may work to strengthen food regulation across borders – has received less attention. Our hypothesis is that exporting countries with high domestic food regulation standards – due to domestic public health concerns and pressure rather than trade concerns – should be interested in encouraging importing countries to strengthen their food regulation, for the sake of protecting their exports from competition from third countries with less stringent food regulation. In our view, the ongoing Cambodian food law reform and the USA active monitoring thereof hints at such dynamic.

After describing the context in which REPLACE came into being, we will explore the example of the USA monitoring Cambodian food law reform, with a view of raising some preliminary considerations about four key issues, setting the ground for further research. First, we will explore how this hypothetical dynamic could work. Who is – or would be – protecting whom from what? Second, we will discuss some procedural fairness considerations. Is it, regardless of the impact of our hypothetical dynamic, fair that the public health promotion of people living in one country is influenced by decisions taken by parliaments in other countries? Third, we will explore alternative mechanisms that could serve the aims of the WHO REPLACE action package. Finally, we will briefly discuss the relationship between REPLACE’s aims and international trade and investment law, providing additional arguments for encouraging international regulatory cooperation, rather than relying on trade dynamics promoting a ‘race to the top’.

### *Trans*-fat in the global fight against non-communicable diseases and REPLACE

NCDs are a leading cause of death. They kill around 41 million people every year, equal to 71% of all human deaths globally [5]. NCDs affect people of all ages and countries, but are rampant in low- and middle-income countries, where three-quarters of all NCD-caused deaths are now happening [5]. The fight against NCDs is a top public health priority both for the United Nations (UN) and WHO. The UN 2030 Agenda for Sustainable Development set the target of reducing the mortality rate from NCDs by one third via prevention and treatment (Sustainable Development Goal n. 3, Target 3.4) [6]. Platform 2 of the WHO General Program of Work 2019–2023 aims to accelerate action on preventing NCDs by tackling four main risk factors, i.e., tobacco use, alcohol use, unhealthy diets, and physical inactivity [4].

Cardiovascular diseases (e.g., heart attacks) are responsible for most premature NCD-related deaths, the other causes being cancer, chronic respiratory diseases, and diabetes [7]. Like all NCDs, cardiovascular diseases (CVDs) result from a combination of genetic, physiological, environmental, and behavioural factors [8]. Modifiable behavioural factors play a significant role in the risk of CVDs. Unhealthy diets contribute to rising blood pressure, blood glucose and lipids, and obesity, all of which are significant metabolic risk factors [8].

In particular, the intake of *trans*-fat elevates the level of Low-Density Lipoprotein (LDL) cholesterol in the blood. Increased levels of LDL - ‘bad’ - cholesterol in the blood raises the risk of CVDs [9]. When elevated to > 1% of total energy intake, consumption of *trans*-fat increases risks of coronary heart disease (CHD) events and mortality [1]. *Trans*-fat intake is estimated to cause more than half a million deaths from CHD every year globally [1].

Most *trans*-fat in foodstuff is artificial. It is manufactured by adding hydrogen to vegetable oil, which transforms this latter into a solid fat (at room temperature) [1]. When this hydrogenation process was invented, food producers became aware that industrially produced *trans*-fats had several commercial advantages, if compared with fats from animal origin. Artificial *trans*-fat is cheaper and does not go rancid for months [10]. Artificial *trans*-fat is also perceived as somewhat ‘cleaner’ than animal fats. Therefore, food companies soon became keen to use artificial *trans*-fat in foodstuff [10]. Industrially produced *trans*-fatty acids are now widespread in snacks and baked and fried foods.

However, a study published by the *New England Journal of Medicine* in 1990 by Mensink and Katan revealed that artificial *trans*-fats could increase LDL cholesterol [11]. When subsequent studies confirmed this hypothesis, national and international public health national and international bodies and organisations and the general public became increasingly aware of the risks associated with eating artificial *trans*-fats.

Although *trans*-fat intake has decreased over the last ten years in many European countries, and while its average consumption is currently relatively low, different socioeconomic groups show unequal intake levels, affecting the poorest people most (since food made with *trans*-fat is cheaper) [1]. Reducing consumption of *trans*-fat is thus a matter of health equity, i.e., a matter of addressing avoidable health inequality.

Building on the wide scientific consensus about the negative effects of industrially produced *trans*-fat on human health, as well as on country-specific regulatory initiatives, WHO developed a specific action plan to reduce industrial *trans*-fat consumption. In line with its General Program of Work 2019–2023 [7], WHO released the REPLACE action package in May 2018 [1], calling on governments to remove *trans*-fats from the global food supply by 2023. The action package claims that it is an effective and efficient way to fight CVDs [1]. It holds the view that eliminating industrially produced *trans*-fat is economically, politically, and technically viable, claiming that any country can quickly replace hydrogenated oils with healthier ones, preferably containing polyunsaturated fats.

REPLACE provides countries with tools to eliminate artificial *trans*-fats from their national food supply chains. In particular, REPLACE contains a six-step action package for the global removal of *trans*-fat in a prompt, complete, and sustained way. The six REPLACE steps include:

- **RE**view dietary sources of industrially produced *trans*-fat and the landscape for required policy change;
- **P**romote the replacement of industrially produced *trans*-fat with healthier fats and oils;
- **L**egislate or enact regulatory actions to eliminate industrially produced *trans*-fat;
- **A**ssess and monitor *trans*-fat content in the food supply and changes in *trans*-fat consumption in the population;
- **C**reate awareness of the negative health impact of *trans*-fat among policy-makers, producers, suppliers, and the public;
- **E**nforce compliance with policies and regulations.

The REPLACE action package recognises that the most effective tool to reduce artificial *trans*-fat in the food supply and individual consumption is legislative or other regulatory action aimed to limit or prohibit industrially produced *trans*-fats [1].

### National regulation and international market dynamics: could there be upward pressure?

REPLACE contains a review of recent regulatory initiatives targeting *trans*-fat in food and assesses their effectiveness. Denmark pioneered regulatory efforts against *trans*-fat by implementing legislation in 2003, which effectively limited artificial *trans*-fatty acids to 2% of total fat in all marketed foodstuff, including imported foods and restaurant meals [1]. Other countries (or cities) used an approach based on voluntary measures or self-regulation: e.g., Canada, the Netherlands, the UK, and New York City (NYC). The voluntary approach produced mixed results. For instance, while Canada secured major reductions of artificial *trans*-fatty acids in its national food supply, the measures implemented in NYC did not prove equally successful. Canada implemented a structured approach featuring a strong surveillance mechanism, public statements about progress, and promised – or threatened with – mandatory regulation if the industry would fail to meet the targets [12]. In contrast, NYC adopted a purely voluntary intervention which did not yield substantial results, which induced the local government to move towards mandatory regulation [13].

REPLACE also includes references to the role of international law in the spread of anti-*trans*-fat regulations. In 2007, the Pan American Health Organization (PAHO) set up a Task Force to foster the vision of Trans-fat Free Americas by legislative action. Several American countries took different actions [1]. For instance, Argentina adopted and implemented legislation to reduce industrially produced *trans*-fat, including the mandatory labelling of *trans*-fatty acids in food in 2006 and the limitation of industrially produced *trans*-fat to 2% of total fats in vegetable oils and below 5% of total fats in other foods [14].

REPLACE does not include reference to more recent regulatory actions that are still underway and the effectiveness of which is still to be assessed. However, in the context of comparing regulatory approaches to *trans*-fat reduction, the recent regulatory developments undertaken by the USA are important. The US Food and Drug Administration (FDA) is committed to eliminating industrially manufactured *trans-*fats in processed foods. In 2015, the FDA declared that Partially Hydrogenated Oils (PHOs), which represent the primary origin of artificial *trans-*fat in the food supply, were no longer “Generally Recognized as Safe” [15]. This implies that *trans*-fat is no longer exempted from regulatory oversight by the FDA. The FDA banned PHOs starting from 18th June 2018, in principle, but granted a deadline extension (until the 1st of January 2020) to allow manufacturers to distribute products made before the ban [15]. With this extension, the FDA tried to strike a balance between the public health objective (the elimination of *trans*-fat from the food supply) and the economic interests.

Some low- and middle-income countries are preparing legal or regulatory action too. On 16th of July 2015, the Cambodian Ministry of Commerce circulated a draft of the country’s first food regulation law, setting up a Food Safety Authority responsible for safeguarding Cambodian consumers and making sure that food exports are in line with international standards [16]. The Cambodian draft food law is the result of a collaboration between the UN Food and Agriculture Organisation (FAO) and several ministries, in consultation with national and international jurists and representatives from the industry [17]. The draft law lists several sanctionable violations, such as the sale of foodstuff containing substances that are harmful to health [17].

The draft law reflects the Cambodian government’s ambition to make the country an important food exporter. The government stated that strict food law is essential to win the trust of international consumers [17]. Indeed, implementing a food law is cheaper than obtaining international certification [18]. However, Cambodian authorities have not finalised the food law yet, which is languishing in its draft stage [18]. The national industry is pushing the government to speed up the approval of the law, which is deemed necessary to improve food exports [18].

The US Department of Agriculture’s Global Agriculture Information Network (GAIN) is actively monitoring the progress of the Cambodian draft food law [16]. GAIN provides information about other countries’ policies that may have an impact on the US agricultural sector [19]. How could Cambodian food affect US economic interests?

As mentioned above, the Cambodian food industry hopes that stricter national food law will increase its market abroad. We do not think this is a major issue for the US, given that the Cambodian food industry is still a small player.

We can speculate that, given the FDA ban PHOs discussed above, the US agricultural sector is looking for an ‘alternative market’ in other parts of the world. If that is the case, we could expect some pressure from the USA on the Cambodian government to adopt weak (or no) regulation on *trans*-fats.

However, the interests of the US food industry may be somewhat different from the interests of the US agricultural sector (even if these two sectors are at least partially overlapping). Given the stricter standards now applied in the US, similar strict regulations in Cambodia would not be a barrier for US companies to export to Cambodia. Only if Cambodia would adopt even stricter regulation it would hinder US food exports to the South-Eastern Asian country. Furthermore, the US food industry would probably welcome stricter food regulation in Cambodia – we mean stricter than it now is, not stricter than the US regulation – as it would hinder export to Cambodia from other countries with weaker standards. If Cambodia adopts the same level of regulation as the US, the US food sector would have a comparative advantage, in a context of US food exports to Cambodia facing significant competition from the Asian region [20]. On balance, the US may be interested in Cambodia increasing its standards and regulations to the level of the US market – not higher, not lower.

To be clear, we are formulating a hypothesis here: further research is required to confirm it (or not). We think such research would be valuable. However, we think such research would have to consider some additional issues. If there is some upward pressure – powerful states encouraging others to adopt more stringent regulation – we should not be naive about who is trying to protect whom, and why. National regulation in the USA seems designed to protect or promote the health of US citizens. Encouragement from the USA towards Cambodia adopting stricter regulation would – in our hypothesis – be designed to protect the interests of the US food industry. That raises additional concerns.

### Exporting *trans*-fat restrictions through trade dynamics: some practical and principled considerations

Our hypothesis can be captured and simplified, using a three-country model. In this model, a country (A) that has relatively strict *trans*-fat restrictions at home could have an interest in ‘exporting’ its regulations to a country (B), so that export of foodstuff from A to B is not affected by competition from country (C) where no or rather weak regulation applies. Indeed, if B increases its regulation to Country A’s level, Country C’s food manufacturing sector will face significant adjustment costs to continue exporting to Country B, while Country A’s food industry could take the market share of Country C’s food industry. This dynamic could contribute to the diffusion of anti-*trans*-fat policies.

Literature documents how exporting countries often pressure importing countries via litigation – and other so-called ‘regulatory chill’ efforts – to lower (or not to increase) regulation [21]. Our discussion of the Cambodian draft food law suggests that in some situation, exporting countries may have an economic interest in what we may call ‘regulatory boost’ efforts. It can be difficult to find direct evidence of such transnational lobbying activities. While international trade disputes provide plausible evidence of ‘regulatory chill’ efforts, they are unlikely indicators of ‘regulatory’ boost efforts. However, even without evidence of the US lobbying the Cambodian government to tighten *trans*-fat regulation, the situation allows imagining that the phenomenon of a ‘race to the top’ – as it is called in policy diffusion literature [22] – occurs.

Such diffusion dynamics deserve being researched deeper than they currently are. However, we think that they also deserve a scholarly discussion that goes beyond the empirical question of whether it is truly happening. A regulatory ‘race to the top’ would have positive public health outcomes, since they would tighten *trans*-fat regulations around the world. But it may be a very slow process, with several setbacks.

Policy diffusion is often a slow process. Furthermore, the policy diffusion speed is slower than usual when policies have a ‘potential for conflict’, i.e., when they have clear distributive effects, with evident winners and losers [23]. This would also apply to *trans*-fat restrictions. In our three-country model: Country A gains are Country C’s losses. And within Country B we can easily imagine winners and losers.

At a principled level, a ‘race to the top’ is problematic as it would represent a form of ‘*élite* multilateralism’ [24], which raises concerns in terms of representativeness, inclusiveness, and accountability. Even if the health outcomes in Country B would improve, they would improve because of a decision to promote the health of the inhabitants of Country A first, followed by a strategy to protect the economic interests of Country A. At least in terms of procedural fairness, this would be problematic [25]. Furthermore, it would strengthen the case of the potential ‘losers’ within Country B trying to oppose the policy diffusion.

### Alternative solutions: international regulation of *trans*-fat restrictions

As discussed above, the combination of regulatory restriction with domestic public health intentions and trade dynamics as a potential solution for reducing *trans*-fat in food globally has severe limitations. We believe that alternative mechanisms should be explored at the same time. Further research could draw useful lessons from the experience of the Framework Convention for Tobacco Control (FCTC) [23, 26, 27]. Concluded at the initiative of the WHO, the FCTC urges parties via binding commitments and non-binding guidelines to implement evidence-based tobacco control measures. Since the FCTC came into effect, an increasing number of countries around the world has adopted such policies, also thanks to the efforts of the Framework Convention Alliance, a network of global health NGOs advocating for tobacco control, which consolidated while the FCTC was negotiated and is now a recognised global tobacco control actor [23]. These developments occurred after and arguably as a result of decades of research and advocacy against tobacco use, which gradually made tobacco control a widely accepted norm [23].

In a similar fashion, the issuance of the REPLACE action package after decades of research and advocacy activities against *trans*-fat in food signals that, in principle, important international actors (namely, member states of the WHO) broadly agree that *trans*-fat restrictions are necessary. Drawing from the FCTC experience, the next step in the fight against *trans*-fat in food could be the development of a deeper institutional framework to promote such restrictions globally. Given that WHO is mandated to promote negotiations and achieving consensus about health policy frameworks [27], it could facilitate the diffusion of *trans*-fat restrictions by promoting an *ad hoc* convention (a Framework Convention for *Trans*-fat Replacement, FCTR) to codify such standards (e.g., gradual *trans*-fat ban in food), possibly assisted by a Secretariat in charge of supporting anti-*trans*-fat norm diffusion from the international to the national level [23]. A WHO-supported international treaty would ensure participatory negotiation in the global fight against *trans*-fat, which could ensure a procedurally fairer alternative to lobbying activities from powerful countries. The bargaining power of powerful countries can be expected to be lower in multilateral settings than in bilateral ones, since, in the former, weaker countries sharing similar interests can join their forces to advance their interests *vis-à-vis* powerful counterparts*.* Given the high potential for conflict and the practical implementation difficulties for poorer countries that are inherent in such restrictions, a balanced international arrangement could foresee a gradual removal of *trans*-fat from the food supply, coupled with a system of financial and technical support for low- and middle-income countries.

However, the development of an *ad hoc* convention targeting *trans*-fat may not be currently politically feasible for two reasons. First, there is no clear evidence of an appetite for such a convention in the international community. Indeed, the REPLACE action package does not mention a framework convention among the regulatory tools that could be used to restrict *trans*-fat use in the global supply chain. Second, governments may not be interested in spending energy negotiating one on a single food-additive. Rather, they may tend to consider general framework conventions as more efficient tools to tackle global health issues. After all, the FCTC experience suggests that developing a framework convention may require years of groundwork to build political and social consensus. Nevertheless, an ‘issue network’ or ‘transnational advocacy network’ [23] similar to the pro-tobacco-control Framework Convention Alliance could play a role in anti-*trans*-fat norm diffusion globally. WHO could facilitate a coalition among NGOs with global reach researching and advocating for a *trans*-fat ban in the food supply. Further research on the topic could draw useful lessons for the global fight against *trans*-fat from the experiences of tobacco control transnational advocacy networks and other epistemic communities involved in other product-based regulatory issues.

As an alternative, the WHO could contribute to the diffusion of such restrictions by including a *trans*-fat ban in the *Codex Alimentarius*, a set of non-binding, yet internationally recognized standards, guidelines, and recommendations concerning food production and food safety, established by FAO and WHO.

### The quest for policy coherence between *trans*-fat restrictions and trade and investment commitments: risks and opportunities

Further research on anti-*trans*-fat policy diffusion should also cover potential challenges and opportunities coming from international trade and investment law. *Trans*-fat restrictions could be at odds with international law commitments to trade liberalisation and investment protection. The banning of import of products containing *trans*-fat could lead to a trade challenge under Technical Barriers to Trade (TBT) rules agreed under the World Trade Organisation (WTO) TBT Agreement, or under a bilateral or regional Free Trade Agreement (FTA) that confirms and/or expands upon such rules. Indeed, TBT disputes are rather frequent [2, 28]. However, in a potential TBT challenge filed by an export country, a defendant country could invoke the REPLACE package and, *a fortiori*, the commitments and guidelines under a hypothetical FCTR in defence against such a challenge. A hypothetical FCTR could provide a sound legal basis for increased anti-*trans*-fat restrictions in import countries [27]. Likewise, since it is recognised by the WTO as an international reference standard for food dispute resolution, a revised *Codex Alimentarius* including a *trans*-fat ban in food would likely prevent similar disputes, thus facilitating norm diffusion internationally [29].

In a similar fashion, an increased institutionalisation and standardisation of anti-*trans*-fat measures could also prevent international disputes under bilateral trade and investment agreements. Although, contrary to tobacco control, food-related disputes under FTAs are scarce, this does not imply that FTAs are irrelevant when it comes to *trans*-fat ban. The relative scarcity of food-related disputes under FTAs is more likely due to the fact that food regulations are ‘a relatively new area of activity’ [29]. Under Investor-State Dispute Settlement rules, which are included in many new-generation FTAs, foreign investors can challenge a governments’ regulatory actions that they believe to reduce the value of their investment, which could extend to claims of compensation [30, 31]. It is not difficult to construct an argument how a *trans*-fat ban might reduce the value of recent investments in national food production and supply chains. The risk of foreign investors suing for compensation could be enough to induce ‘regulatory chill.’ However, investment protection rules under FTAs do not prevent regulatory innovation (and tightening) *per se*, provided that the new regulation is fair, equitable, and non-discriminatory. In the context of a potential international trade dispute, defendant countries, as well as WHO and public health NGOs as *amici curiae*, could refer to REPLACE and possibly a revised *Codex Alimentarius* for an authoritative and evidence-based defence. In any event, it is crucial for governments not to induce foreign investors to have ‘legitimate expectations’ that such restrictions will not be implemented. Rather, governments should preserve their policy space by clarifying that foreign investors cannot expect the host country not to implement public health nutrition measures, such as a *trans*-fat ban.

At the same time, the institutionalisation and standardisation of the global fight against *trans*-fat could help exploit the potential of bilateral trade and investment frameworks to further contribute to anti-*trans*-fat norm diffusion. While international trade rules and principles both in WTO rules and FTAs are structurally designed to fight forms of protectionism, i.e., to decrease unreasonable or discriminatory standards and regulations, new-generation FTAs also contain regulatory cooperation sections, aimed to foster the harmonisation of standards and regulations, which, albeit to a limited extent, can also contribute to increasing these latter. In addition, an increasing number of FTAs contains regulatory coherence and sustainable development rules, according to which regulatory measures must be reviewed to advance the achievement of domestic and sustainable development policy objectives. By invoking evidence-based WHO instruments and guidelines, an alliance of global NGOs under the remit of the WHO could yield useful results within or on the side of regulatory cooperation, regulatory coherence, or sustainable development *fora* under new-generation FTAs (e.g., Chapters XXI and XXII of the Canada-EU Comprehensive Economic and Trade Agreement (CETA)). The main limitation of such CET chapters is that they establish unenforceable review mechanisms only, which are not subject to trade dispute resolution. Therefore, advocacy within such *fora* could be normatively reinforcing, but not legally binding in any way. The same applies to the recent US-Mexico-Canada Agreement (USMCA) of 2018, under which the parties shall enable any ‘interested person’ to provide inputs about regulatory cooperation and coherence (Arts. 25.8 and 28.9, n. 3, USMCA). Public health nutrition NGOs residing in one of the other countries that are party to the agreement (i.e., in Canada, Mexico, or the USA) could use such *fora* to advocate for a regulatory harmonisation that is in line with global health policy objectives. To this end, while the interest of exporting countries in increasing the level of regulations in importing ones seems incapable of improving public health alone, public health advocates could use it as an economic and political leverage for their advocacy endeavours.

In addition, competition law rules under FTAs could be used to react to harmful commercial strategies aimed to chill *trans*-fat regulations. Indeed, transnational corporations might use their economic power to deter weaker countries from adopting stricter standards, e.g., by threatening to divest and withdraw their food products from the market. Competition law enforcement could help react to such strategies, which could amount to anticompetitive agreements or abuses of dominant positions. International organisations have already considered the use of competition law for public health purposes [32]. As for future agreements, public health NGOs and World Health Assembly delegations could ask of their government negotiators to have more input into new trade or investment negotiations, making sure that health nutrition standards are not considered as protectionism. Indeed, early engagement with trade policymakers could be beneficial to ensuring full protection of public health regulatory policy space within new treaties [29].

## Conclusions

An aspect overlooked by the existent trade and health literature, we hypothesised how some countries might be interested in encouraging other countries to adopt their higher level of regulation, for the sake of discouraging competition from third countries with less stringent regulation. We formulated some preliminary considerations about four key issues, setting the ground for further research on the topic.

First, we explored the plausibility of the hypothesised dynamic. We concluded that, in some circumstances, some countries may indeed try to promote their own interests by encouraging other countries to adopt stricter regulation.

Second, we discussed some practical and principled considerations. Our preliminary analysis suggests that the diffusion of anti-*trans*-fat policy through trade dynamics can be expected to be lengthy, given the high potential for conflict, raises concerns about procedural fairness, while the latter may reinforce the first.

Third, we explored alternative mechanisms that could contribute to *trans*-fat restriction globally in a more effective and cooperative manner. Revised *Codex Alimentarius* standards at the initiative of WHO could be key to codifying such norms and facilitate diffusion from the international to the national level. Deeper institutionalisation*,* e.g., through a WHO-supported framework convention and an *ad hoc* Secretariat, could also be useful, but currently it does not seem to be politically feasible. A ‘transnational advocacy network’ of global health nutrition NGOs under the remit of the WHO could help build political and social consensus regarding the need for stronger *trans*-fat restrictions. Further research on this issue could benefit from the appropriate consideration of other product-based regulatory experiences.

Fourth, we discussed the relationship between these alternative cooperation mechanisms and international trade and investment law. On the one hand, the REPLACE action package and increased institutionalisation of *trans*-fat restrictions (e.g., revised *Codex Alimentarius* standards) could provide a sound basis for defences against challenges under multilateral and bilateral trade agreements. On the other hand, new-generation FTAs could provide for *fora* that may be used to advocate for anti-*trans*-fat policy diffusion, namely, those established under regulatory cooperation, regulatory coherence, sustainable development, and competition policy sections. Further research on this issue is also needed to develop a more thorough analysis of potential opportunities and anticipated challenges related to international economic law.

## Data Availability

Not applicable.
